# Objective Assessment of Ideomotor Limb Apraxia Using Markerless Computer Vision and Machine Learning: A Proposed Framework and Validation Design

**DOI:** 10.7759/cureus.114763

**Published:** 2026-08-18

**Authors:** Alireza Minagar

**Affiliations:** 1 School of Cybersecurity and Information Technology, University of Maryland Global Campus, Adelphi, USA

**Keywords:** apraxia, computer vision, gesture, ideomotor apraxia, kinematic analysis, machine learning, neurological assessment, pose estimation, sensor fusion, wearable sensors

## Abstract

Ideomotor limb apraxia is a higher-order disorder of learned, skilled movement that cannot be adequately explained by elementary weakness, sensory loss, ataxia, extrapyramidal dysfunction, impaired comprehension, or lack of cooperation. It is common after left hemisphere stroke and occurs in several neurodegenerative diseases. Quantitative kinematic study of apraxic movement is well established in the research literature, and standardized clinical instruments such as the Test of Upper Limb Apraxia and its bedside screen have improved the reliability of the examination. What remains missing is a bridge between these two bodies of work: an approach that uses only ordinary video, requires no laboratory motion capture, and maps recovered movement features onto a clinically interpretable profile of apraxic errors for routine bedside or remote use. This report advances the hypothesis that markerless computer vision, combined with machine learning trained against blinded expert consensus, can serve as an objective measurement adjunct for ideomotor limb apraxia: quantifying selected spatial and temporal features of gesture production, profiling error dimensions at the level of individual trials, and, in a more limited way, supporting detection and grading. The hypothesis is deliberately framed as a measurement adjunct rather than an autonomous diagnostic system. This report sets out the clinical rationale, the narrowed novelty claim, the proposed framework and its two analytical levels, the outcomes that would be predicted if the hypothesis holds, a validation design whose central feature is a control group of neurologically affected patients without apraxia, and the substantial limitations that bound the proposal.

The framework specifies, as prespecified comparisons rather than as assumptions, alternative pose estimation families including transformer-based estimators, optional inertial augmentation with filter-based video inertial fusion, and alternative classifier architectures. Each is presented as a question the validation study should answer. This report presents a theoretical framework and a validation design only. No empirical patient data were collected, no patients were recruited, and no results are reported.

## Introduction

Apraxia is one of the classical disorders of higher cortical function. Hugo Liepmann framed the modern concept more than a century ago, and the intervening decades have produced a detailed understanding of its anatomy, its cognitive architecture, and its dissociations. Apraxic movement has also been studied quantitatively for decades. Three-dimensional computer graphic analysis of apraxic gestures was reported as early as 1990 [1], and kinematic analyses have since documented characteristic spatial and temporal abnormalities in apraxic reaching and imitation [2,3]. The disorder is therefore not accessible only through subjective impression; it has measurable motor manifestations.

Two developments make it worth revisiting this problem now. First, markerless pose estimation can recover body and hand landmarks from ordinary video: open source frameworks such as OpenPose and Google's MediaPipe now provide real-time body and hand landmark estimation from a single RGB camera [4-6], and these tools have been applied to human health and movement assessment across many settings [7]. Computer vision has been shown to yield clinically meaningful upper limb kinematic metrics using only modest cameras and open source tools [8]. Second, machine learning routinely classifies movement patterns in adjacent neurological, rehabilitation, and skill assessment settings [9].

Two gaps in this recent literature matter for the present proposal. The prior kinematic work on apraxia relied on laboratory motion capture or specialized recording, which is why it never entered routine clinical use. Conversely, the recent explosion of markerless computer vision in stroke has focused almost entirely on elementary motor impairment, quantifying paresis, range of motion, and compensation during reaching or automating scales such as the Fugl-Meyer Assessment [10], rather than the higher-order cognitive motor planning deficits that define apraxia. The question this report raises is whether the informative measurements can now be approximated from ordinary video and organized around the way clinicians already classify apraxic errors, which is a target that neither body of work has addressed.

The proposal is developed here as an explicit and bounded hypothesis. The claim is not that apraxia can be diagnosed automatically or that expert assessment can be replaced. The claim is narrower and correspondingly more defensible: that a markerless, video-based system can act as an objective measurement adjunct that quantifies selected features of apraxic gesture production and profiles error dimensions, complementing rather than supplanting the clinician. The sections that follow define apraxia and describe how it is currently assessed, identify the specific gap, state the hypothesis in testable form, describe the framework and its limits, set out predicted outcomes and what would refute them, propose a validation design built around the appropriate control group, and set out the limitations.

Background: the clinical problem

Defining Ideomotor Limb Apraxia

A clinically careful definition is important, because the entire proposal depends on distinguishing apraxia from disorders that can mimic it. Limb apraxia is best described as a higher-order disorder of learned or purposeful movement that cannot be adequately explained by elementary weakness, sensory impairment, ataxia, extrapyramidal dysfunction, impaired comprehension, or lack of cooperation [11]. The phrase cannot be adequately explained is deliberate: many apraxic patients also have hemiparesis, sensory loss, or aphasia, and the clinical task is to judge whether those coexisting deficits sufficiently account for the abnormal movement. Contemporary accounts describe limb apraxia as a spectrum of higher-order motor disorders associated chiefly with left hemisphere stroke and with neurodegenerative disease, rather than as a single unitary entity.

The classical division into ideomotor, ideational, and limb kinetic apraxia remains widely recognized but is not applied uniformly, and modern work often characterizes impairment by task, processing component, and error pattern rather than assuming discrete syndromes. For clarity, and to avoid resting the argument on contested subtype terminology, the target of this report is operationalized as follows. In this report, ideomotor limb apraxia refers to impaired production of learned or novel gestures under command or imitation, characterized predominantly by spatial and temporal execution errors that are not adequately explained by elementary motor, sensory, language, or attentional deficits. This form is the natural first target for a movement-based method, because its signature lies in how a gesture is executed rather than in what the patient knows about the action.

How Apraxia Is Currently Assessed

It would be inaccurate to say that no reliable standardized assessment exists. The Test of Upper Limb Apraxia, or TULIA, assesses gesture production across imitation and pantomime for non-symbolic, intransitive, and transitive gestures, using a six-point scoring method across 48 items, and it demonstrated generally good to excellent internal consistency and interrater and test-retest reliability in a validation sample of stroke patients and healthy controls [12]. A 12-item bedside reduction, the Apraxia Screen of TULIA, was derived and validated for rapid screening [13]. Other batteries have similarly standardized the examination [14].

These instruments improve reliability, but they share two features that define the gap addressed here. First, scoring rests on human observation of the movement, whether live or from video, and therefore inherits the variability of human judgment; even well-constructed scales report agreement that is good for summary scores but more modest at the level of individual items. Second, the output is an ordinal score rather than a continuous, physically interpretable measurement of the movement itself. A clinician can record that a gesture was moderately impaired, but not that the hand deviated from the target trajectory by a specific margin or that the movement contained a measurable excess of submovements. A more accurate statement of the problem is therefore not that standardized assessment is absent, but that standardized scores remain based primarily on observer judgment and do not provide continuous, instrument-derived measurements of movement geometry, timing, coordination, or trial-level error probability.

The Measurable Signature of Apraxic Movement

That apraxic movement differs from normal movement in physically measurable ways is well established. The first three-dimensional analysis of apraxic gestures showed quantifiable differences in the movements of apraxic patients compared with controls [1]. Kinematic analysis of movement imitation demonstrated that apraxic trajectories differ from those of controls [3]. Crucially for the present argument, kinematic analysis of aiming movements showed spatial deficits specific to ideomotor limb apraxia, and, by including left-hemisphere-damaged patients without apraxia as a comparison group, demonstrated that these deficits were not merely a consequence of left hemisphere damage as such [2]. Apraxic errors have long been described along recognizable dimensions, commonly grouped as spatial, temporal, content, and omission errors [15]. The raw material of an objective measure therefore exists conceptually: clinicians are, in effect, perceiving spatial and temporal deviations and classifying error types, and those deviations are the quantities that motion analysis captures.

The Gap and the Novelty Claim

Because quantitative and computerized assessment of apraxia already exists, the novelty of this proposal must be stated narrowly and precisely. It would be wrong to claim that objective or quantitative assessment of apraxia has not been attempted; the work cited above shows that it has. The prior studies, however, relied on laboratory-based motion capture, specialized instrumentation, or manual computer graphic reconstruction, and they typically quantified selected kinematic variables in small research samples. None of this entered routine clinical practice, precisely because the methods were not deployable at the bedside.

At the same time, applying markerless computer vision to upper limb movement in stroke is by itself no longer novel; that literature is already substantial. Its focus, however, has been elementary motor impairment rather than praxis. A model that merely showed that machine learning can analyze upper limb movement would be derivative. The defensible novelty claim is therefore narrower and lies in the intersection: no externally validated framework appears to have been established that uses only markerless video from ordinary cameras, maps recovered movement features specifically onto the spatial and temporal error dimensions of an established apraxia scale rather than onto paresis or range of motion, and is validated against blinded expert consensus for routine bedside or remote use. This claim should be confirmed by a formal, reproducible literature search before submission, and qualified throughout as a probable gap rather than an established absence. The contribution, if the gap holds, lies not in any single technique, since a pipeline of video, pose estimation, feature extraction, and supervised classification is now a standard architecture, but in the clinical representation: the task protocol, the operational definitions of each error dimension, the explicit separation of apraxia from elementary motor impairment, the multidimensional output, and validation across patients, raters, tasks, and recording conditions.

The technical comparisons described in the next section, between pose estimation families, between video-only and sensor-augmented input, and between classifier architectures, are methodological choices within that framework. None of them constitutes a novelty claim in its own right, and none should be presented as one. Adopting a newer model class is not a scientific contribution; establishing whether it changes the clinical measurement is.

## Technical report

The hypothesis

The hypothesis can be stated as follows. Markerless computer vision applied to standardized gesture tasks, combined with machine learning trained against blinded expert consensus ratings, can function as an objective measurement adjunct for ideomotor limb apraxia: it can quantify selected spatial and temporal features of gesture production as continuous, physically interpretable measures; it can profile the dimensions of apraxic error at the level of individual trials; and it can support, though not by itself establish, the detection and grading of apraxia.

Two claims are embedded here and can be tested independently. The first is a measurement claim: that continuous spatial and temporal features recovered from ordinary video distinguish apraxic from non-apraxic gesture production and track severity. The second is a criterion validation claim: that a model can approximate blinded expert judgments of the same performances. The second must be framed carefully. A model trained on expert ratings can, at best, learn to reproduce those ratings, and agreement with experts does not by itself prove that the model measures the underlying disorder more accurately than the experts do. Expert consensus is therefore best treated as a reference standard for criterion validation, not as unquestioned ground truth, and the measurement claim should be anchored additionally to independent evidence such as functional performance, lesion characteristics, and longitudinal change. Phrases such as accuracy comparable to expert consensus are also underspecified, since they could mean agreement with a consensus label, equivalent sensitivity and specificity, equivalent reliability, or noninferiority to individual raters; these are distinct hypotheses and should be specified separately in any test.

The proposed framework

**Figure 1:**
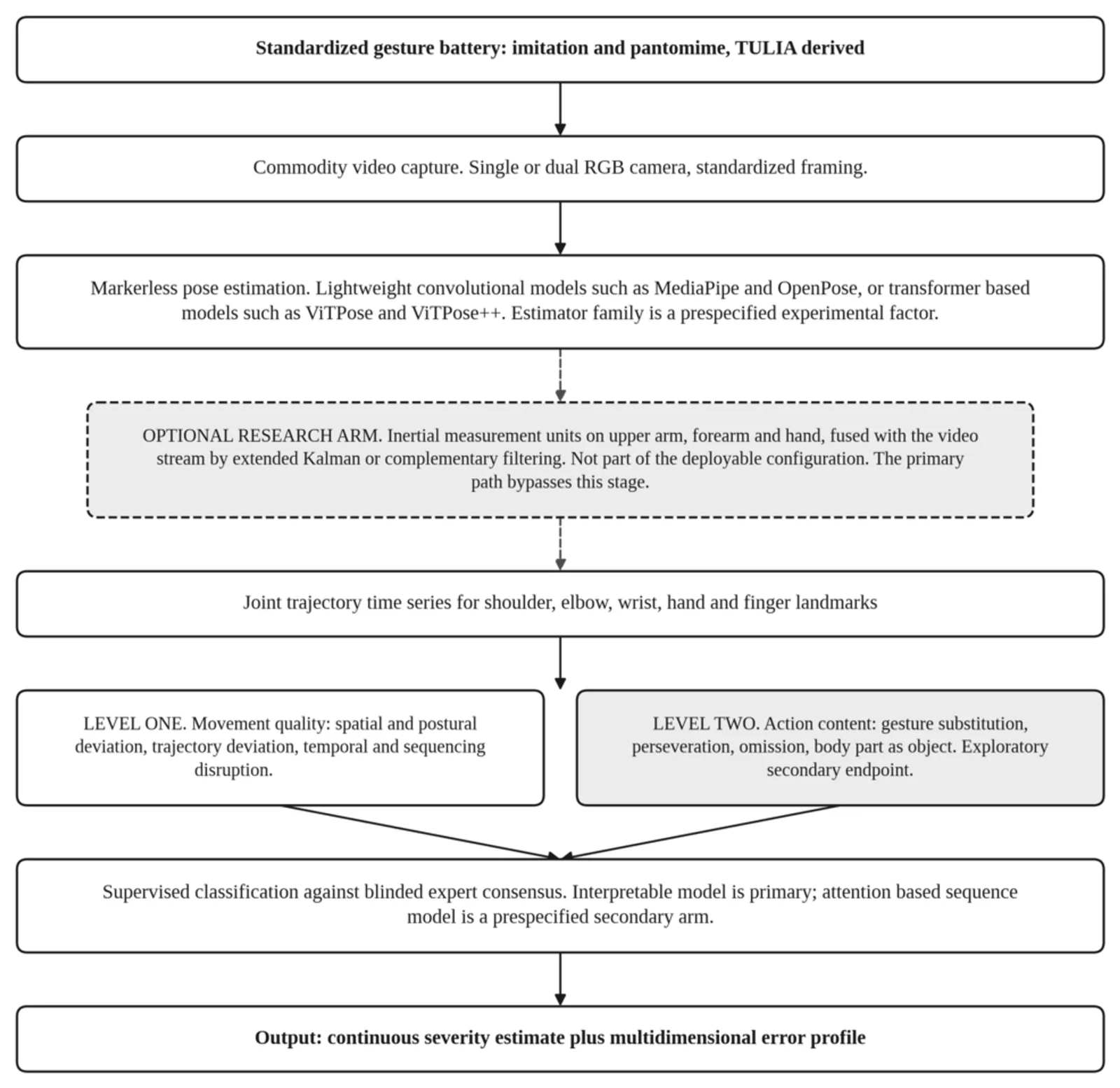
Proposed processing pipeline for markerless assessment of ideomotor limb apraxia Solid outlines mark the video-only path, which carries the clinical claim and is deployable at the bedside or over a telemedicine link. The dashed stage marks optional inertial augmentation with filter-based fusion, included as a research-grade comparison arm rather than as part of the proposed clinical system; the primary path bypasses it. Feature extraction is divided into level one, movement quality, and level two, action content, the latter retained as an exploratory secondary endpoint. Candidate features for each dimension are given in Table 1. TULIA: Test of Upper Limb Apraxia

The proposed system has two analytical levels, a distinction that the critique of a purely kinematic approach makes necessary. Movement quality analysis addresses how a gesture was executed, in spatial and temporal terms. Action content analysis addresses whether the observed gesture matched the requested target. Kinematics can support the first level well and the second only partially, and being explicit about this boundary is part of stating the hypothesis responsibly. Figure 1 shows the complete pipeline, including the optional components described below.

**Table 1:** Apraxic error dimensions, candidate kinematic features, and feasibility from commodity video Level one of the framework comprises the first four rows. Body part as object errors are excluded from the level one feature vector in initial training, for the reasons given in the text, and are treated together with the content and conceptual errors of the final row as exploratory endpoints. Feasibility ratings are prospective judgments, not measured values.

Error dimension	Clinical description	Candidate kinematic feature	Feasibility from single camera commodity video
Spatial and postural	Incorrect limb or hand posture; movement carried out in the wrong plane; hand oriented wrongly relative to the imagined object	Absolute and relative shoulder, elbow, and wrist angles; hand orientation relative to the forearm and to the target plane; endpoint position error against an idealized target	Moderate. Gross joint angles recover reliably; hand orientation in depth is among the least reliable quantities from a single view
Trajectory	Distorted movement path; excursion too large or too small; departure from the expected shape of the gesture	Path length; path curvature; dynamic time warping distance from a normative template gesture; movement amplitude	High for gross limb trajectory under standardized framing
Temporal and sequencing	Slowed or irregular movement; hesitation; fragmentation into submovements; components produced in the wrong order	Movement onset latency; movement time; peak velocity and its timing; number of velocity peaks; pauses and corrections; order of submovement components	High. These are the most robustly recoverable features and carry much of the expected signal
Intersegmental coordination	Decoupling of joint motions that are normally tightly linked during a skilled gesture	Inter joint phase lag; cross correlation between joint angle time series; trial to trial variability	Moderate. Sensitive to keypoint jitter, so smoothing and per trial quality control are required
Body part as object	The hand is used as though it were the tool itself rather than as though holding the tool	Hand configuration descriptors, for example the angle between the index finger and the palm plane, or the distance from the fingertip to the presumed point of tool contact	Low. One candidate implementation among several; depends on exactly the fine hand configuration that single camera estimation recovers least reliably. Excluded from the level one feature vector in initial training.
Content and conceptual (level two)	Wrong gesture produced; semantically related or unrelated substitution; perseveration; omission of a critical component	Not recoverable from trajectory statistics alone; requires comparison of the produced gesture against the requested target and a reference gesture, and may require hand mesh reconstruction or video embeddings	Low. Retained as an exploratory secondary endpoint; the strength of the hypothesis rests on the dimensions above

Video Capture

A patient performs a standardized set of gesture tasks, aligned with a validated battery such as the TULIA, while an ordinary camera records the upper limbs. Reliance on commodity video, rather than marker-based motion capture or specialized depth sensors, is what would make the approach feasible at the bedside and, in principle, over a telemedicine link, and is the feature that distinguishes it from prior laboratory work. Acquisition frame rate is a design parameter rather than an incidental property of the recording, because several of the proposed temporal features depend on it directly. At 30 frames per second the highest frequency that can be represented without aliasing is 15 hertz, which is adequate for movement duration, onset latency, and the counting of well-separated submovements, but marginal for resolving closely spaced velocity peaks and unreliable for any feature derived from the second or third derivative of position, since numerical differentiation amplifies high-frequency noise. Sixty frames per second is therefore recommended for the validation study. Frame rate should be recorded for every session, reported, and entered as a covariate in any multisite analysis. Where recordings are pooled across devices, all trajectories should be resampled to a common rate and filtered with an identical low-pass cutoff before feature extraction, so that a difference in submovement count between sites reflects the movement rather than the camera. The standardized acquisition conditions assumed throughout are set out in the checklist below.

Acquisition conditions should meet the following standards: illumination of at least 300 lux at the participant, frontal and diffuse, with no light source or window in the camera field of view; a plain, matte, non-patterned background contrasting in value with clothing and skin; short-sleeved or close-fitting clothing to above the elbow, plain and contrasting with the background, with watches, bracelets, and rings removed; a fixed tripod with the lens at seated shoulder height, perpendicular to the participant frontal plane, at 1.5 to 2.0 metres; the head, trunk to hip level, and both upper limbs in frame throughout the full range of movement; a minimum resolution of 1920 by 1080 at 60 frames per second, with fixed focus, fixed exposure, and automatic white balance disabled; an armless chair of fixed height with the feet flat and the back unsupported; a 10-second calibration segment at the start of each session, comprising rest followed by one full shoulder abduction and return; and device, frame rate, resolution, illuminance, room, and any protocol deviation logged for every session.

Pose Estimation

A markerless pose estimation model, for example MediaPipe Holistic, recovers the positions of the hands, fingers, and arm joints across the frames of each gesture from a single RGB camera. Video-based pose estimation can yield upper limb kinematic metrics of acceptable accuracy when compared against marker-based reference systems for gross movements [8]. A specific technical challenge must be named at the outset rather than deferred, because it is the principal technical vulnerability of the approach: single camera pose estimation degrades severely under self occlusion, and apraxia pantomime is full of exactly the movements that cause it, since gestures such as combing the hair, brushing the teeth, or saluting bring the hand across the body or in front of the face. The accuracy of pose estimation for fine finger configuration and for hand orientation in depth is therefore a genuine constraint, not a detail, and it should itself be validated against manual annotation or motion capture in a subset rather than assumed. The severity of this constraint is sufficient that a multiple-view configuration is recommended here as the default for the validation study rather than treated as a rare exception. A two-camera arrangement, one frontal and one oblique at approximately 45 degrees at the same height and distance, resolves the majority of self-occlusions that arise when the hand crosses the midline or approaches the face, and it permits either triangulation of landmarks visible in both views or, at minimum, selection of the view in which the pose estimator returns the higher per keypoint confidence. The two streams require synchronisation, which a hardware trigger or a visible and audible marker at the start of each recording provides adequately at the frame rates proposed. The single camera configuration is nevertheless retained as the minimum deployable clinical and telemedicine configuration, since it is the configuration on which the deployability claim rests, and the difference in keypoint stability and downstream classification performance between the single view and two view arrangements should be reported as a prespecified secondary comparison, because that difference bounds what the single camera claim gives up.

Choice of Pose Estimation Model

The pose estimator is a component of the pipeline rather than its conclusion, and several families are now available. Lightweight on-device models such as MediaPipe and BlazePose provide real-time body and hand landmarks on ordinary hardware [5,6]. OpenPose recovers multi-person body and hand keypoints using part affinity fields [4]. High-resolution convolutional architectures such as HRNet preserve spatial precision by carrying parallel multi-resolution representations through the network [16]. More recently, plain vision transformer backbones paired with a lightweight decoder, as in ViTPose and its successor ViTPose++, have achieved strong results on standard human pose benchmarks and scale efficiently across model sizes [17,18]. Because these models treat image patches as tokens and use self-attention, they capture long-range spatial dependencies, which is a plausible advantage for localizing distal joints under partial occlusion or in atypical postures.

That plausibility should not be mistaken for an established clinical advantage. The distinction is stated explicitly because it is the point at which expectation most easily outruns evidence. Benchmark accuracy on large natural image datasets does not transfer automatically to a clinical setting characterized by seated hemiparetic patients, self-occlusion during pantomime, uncontrolled lighting, and modest camera resolution, and no published comparison establishes which estimator family performs best on apraxic gesture. Larger transformer variants also carry a computational cost that may be incompatible with the commodity hardware on which the bedside claim depends. The estimator family is therefore treated here as a prespecified experimental factor rather than a settled choice: at least one lightweight convolutional estimator and at least one transformer-based estimator should be run over identical recordings, with keypoint stability compared against manual annotation or motion capture in a subset, and with the downstream effect on classification reported separately from the keypoint-level effect. If the estimators prove interchangeable for the features that matter clinically, that is itself a useful and reassuring result. Table 2 summarizes the estimator families under consideration and the trade-offs each carries.

**Table 2:** Pose estimation model families considered for the tracking layer Estimator family is treated as a prespecified experimental factor rather than a fixed design choice. No claim is made that any family is superior for this application; establishing that is part of what the proposed validation would test.

Model family	Representative models	Basis	Relevant strengths	Relevant limitations
Lightweight on device convolutional	MediaPipe Holistic; BlazePose	Compact convolutional networks optimized for real time inference on ordinary hardware	Runs on commodity devices; integrated body and hand landmarks; already applied to gesture scoring in clinical cohorts	Lower keypoint precision; degrades under occlusion and atypical posture
Multi person convolutional	OpenPose	Part affinity fields linking detected keypoints into person level skeletons	Established and widely validated; recovers body and hand keypoints together	Computationally heavier; hand keypoints less accurate than body keypoints
High resolution convolutional	HRNet	Parallel multi resolution streams that preserve spatial precision throughout the network	Strong keypoint localization accuracy; well characterized on standard benchmarks	Higher compute and memory cost; two dimensional output still requires lifting for depth
Plain vision transformer	ViTPose; ViTPose++	Vision transformer backbone with a lightweight decoder; image patches treated as tokens with self attention	Strong benchmark performance; long range spatial context that may aid localization of distal joints under partial occlusion; scales across model sizes	Data and compute hungry at larger sizes; benchmark advantage on natural images is unproven for apraxic gesture in a clinical setting

Feature Extraction Mapped to the Error Taxonomy (Level One, Movement Quality)

From the recovered kinematics the system computes engineered features chosen to correspond to dimensions clinicians already use. The most feasible targets from ordinary video are movement onset latency, movement duration, peak velocity, the number of velocity peaks or submovements, path length, trajectory deviation, endpoint error, gross joint angle relationships, pauses and corrections, intersegmental coordination, and trial-to-trial variability. Spatial deviation can be quantified, for example, by the dynamic time warping distance between the patient's hand trajectory and a normative template gesture, and temporal disruption by velocity profile irregularity and movement fragmentation metrics. The emphasis on hand-crafted, clinically motivated features rather than end-to-end deep learning is deliberate: apraxia datasets are small, and training deep networks from scratch on a few tens of patients would invite overfitting and would not be a sound design choice. Spatial and trajectory categories overlap, so a single operational taxonomy with defined, interpretable labels must be adopted even though the labels will not be perfectly mutually exclusive.

**Figure 2:**
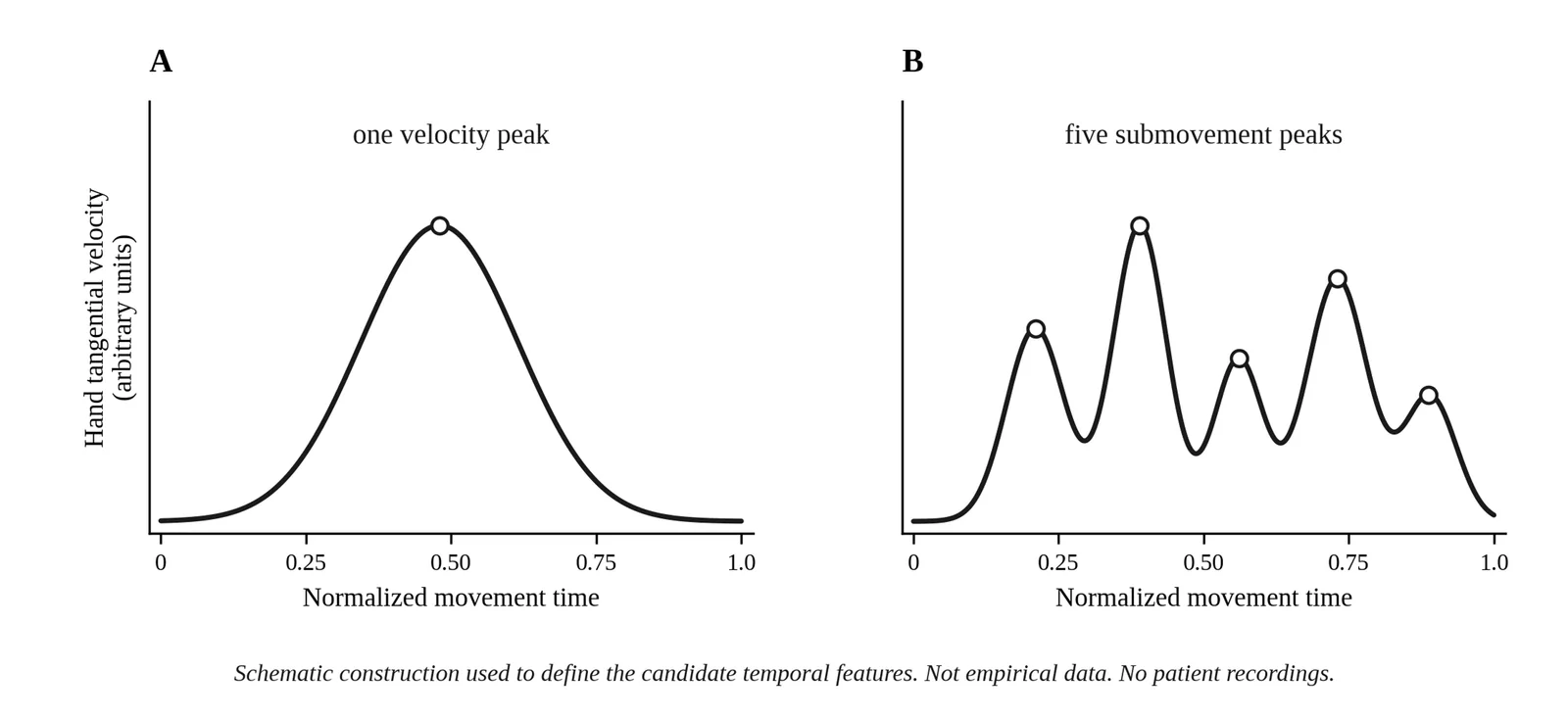
Schematic hand velocity profiles illustrating the temporal features proposed for extraction Panel A shows a single peaked velocity profile of the kind associated with smooth, well planned reaching. Panel B shows a fragmented profile containing multiple submovements, the pattern that the velocity peak count and fragmentation metrics in Table 1 are intended to capture. Open circles mark local velocity maxima. Both traces are schematic constructions drawn to define the candidate features. They are not empirical data, contain no patient recordings, and should not be read as results.

The candidate feature set can be stated more concretely, drawing on variables that laboratory studies have already shown to differ between apraxic patients and controls. Spatial and postural deviation can be indexed by absolute and relative shoulder, elbow, and wrist angles, by hand orientation relative to the forearm or to the target plane, and by endpoint position error against an idealized target. Trajectory deviation can be indexed by path length, path curvature, and departure from a normative template. Temporal and sequencing disruption can be indexed by peak velocity and its timing, the number of velocity peaks, movement time, inter-joint phase lag, and the order of submovement components. Body part as object errors, in which the hand stands in for the tool rather than holding it, might be approached through hand configuration descriptors such as the angle between the index finger and the palm plane, or the distance between the fingertip and the presumed point of tool contact. This last operationalization should be read as one candidate implementation rather than a solved measurement. It is a coarse proxy for what is in practice a categorical clinical judgment, it depends on precisely the fine hand configuration that single-camera pose estimation recovers least reliably, and it will need refinement or replacement against annotated data before it can be relied upon. A practical consequence follows for model training. Because body part as object errors depend on hand orientation in depth and on the fine finger-to-palm angle configuration, both of which single-camera two-dimensional video recovers least reliably, admitting them to the level one feature vector would introduce measurement noise into precisely the analysis whose strength rests on robustly recoverable quantities, and would risk contaminating the learned representation during initial training on a small cohort. Body part as object errors are therefore excluded from the level one feature vector in the initial training of the proposed models and are treated alongside the content and conceptual errors of level two as an exploratory endpoint. They should be reintroduced into level one only where a multiple view or depth resolved configuration, or a validated hand mesh reconstruction, has first demonstrated that the underlying hand configuration can be recovered with acceptable stability against manual annotation. The low feasibility rating in the corresponding row of Table 1 should accordingly be read as the reason for exclusion at level one rather than as a caveat attached to inclusion. Table 1 sets out each error dimension alongside its candidate features and an assessment of how reliably each can be recovered from commodity video, and Figure 2 illustrates schematically the velocity profile features that carry much of the expected temporal signal.

Action Content Analysis (Level Two)

Some apraxic errors concern not how a movement was made but whether the correct action was selected: production of the wrong gesture, semantically related or unrelated substitutions, perseveration, omission of a critical component, use of an inappropriate body part, or an action appropriate to a different object. A conceptual point must be made explicit here, because it bears on the scope of the hypothesis. Spatial and temporal execution errors are the traditional signature of ideomotor apraxia, whereas content and conceptual errors are more closely associated with conceptual or ideational apraxia. Including content errors within a framework aimed at ideomotor apraxia therefore risks a categorical conflation. For that reason this report treats spatial and temporal errors as the primary target, accessible to level one analysis, and content errors as an explicitly exploratory secondary endpoint. Content errors also cannot be identified from trajectory statistics alone; capturing them would require comparison of the observed gesture against the requested target and reference gesture, and may need hand mesh reconstruction, video embeddings, object context, or explicit rule-based representations. Level two is thus a harder, partially open problem rather than a solved one, and the strength of the hypothesis rests on level one.

Machine Learning and Output

The extracted features train a supervised model whose targets are blinded expert consensus ratings, retaining ordinal or probabilistic labels rather than forcing every trial into a binary category. Because the feature set is designed to be interpretable, the model can be kept relatively transparent, which is preferable in a clinical setting to a purely end-to-end approach whose basis cannot be inspected. The output is a multidimensional profile, the spatial, temporal, and, where feasible, content dimensions, rather than a single global label.

Model Class, and the Case for and Against Transformers

Sequence models built on self-attention have performed well on pose-derived time series in adjacent movement recognition tasks, and the architecture fits the present problem in principle: a gesture is a sequence, apraxic errors are often expressed in the ordering and timing of submovements, and self-attention models dependencies across an entire trial rather than only between neighboring frames [19]. Adopting such a model as the default classifier would nevertheless be premature, for a reason that is empirical rather than stylistic. Apraxia cohorts are small, likely fewer than 100 patients, and attention-based models are data hungry and prone to overfitting at that scale; a transformer trained from scratch on a few tens of participants would very probably learn the idiosyncrasies of those participants rather than the signature of the disorder. The design position adopted here is therefore that an interpretable classifier over clinically motivated features remains the primary model, and that a transformer over the same feature sequences is a prespecified secondary arm, admissible only with pretraining or transfer from larger movement datasets, strong regularization, and strictly participant-level evaluation. Whether the transformer outperforms the simpler model is a question the validation study should answer, not an assumption the framework should embed.

Optional Inertial Augmentation and Video Inertial Fusion

Ordinary video is the modality on which the clinical claim of this report rests, because it is what makes bedside and remote use possible. It is nevertheless worth specifying an augmented configuration, both as a research-grade reference and as a way of separating the limits of the sensor from the limits of the hypothesis. Small inertial measurement units placed on the upper arm, forearm, and hand supply high-rate linear acceleration and angular velocity, from which orientation, velocity, and segment trajectory can be derived. A systematic review and correlation meta-analysis of inertial sensing of the upper limb after stroke found that a limited number of wearable sensors yields additional kinematic information across the functional domains examined, with kinematic variables correlating significantly with conventional clinical assessment [20].

The two modalities fail in different ways, which is the argument for combining them. Vision supplies an absolute spatial frame and hand configuration but degrades under occlusion and poor lighting. Inertial sensing is continuous, high rate, and indifferent to occlusion, but it drifts over time and carries no absolute position. A Kalman filter formulation is a principled way to combine the two. In an extended Kalman filter the state vector holds segment positions, orientations, and velocities; inertial data propagate the state between frames through a rigid body process model; and vision-derived landmarks update the state, with observation noise weighted by the per-keypoint confidence score that the pose estimator already returns, so that occluded landmarks are automatically downweighted. A complementary filter is a lighter alternative that blends high-rate inertial signals with low-rate visual correction. Both approaches are established in vision-inertial motion tracking, and both would be expected to reduce drift and to bridge brief losses of visual tracking.

Two Qualifications Attach to This Proposal

Fusion is offered as an optional enhancement and as a comparison arm, not as part of the deployable configuration, since requiring sensors on the limb would forfeit the bedside and telemedicine advantage that motivates the work. And its specific benefit for apraxic error classification, as distinct from general kinematic accuracy, is untested. Its role in the design is therefore to establish an upper bound. If a fused configuration substantially outperforms video alone, the shortfall of the video-only system can be attributed to measurement quality rather than to the hypothesis itself; if it does not, the video-only claim is strengthened.

A proposed validation design

The Critical Comparison Group

The single most important design feature is the comparison group. If apraxic patients were compared only with healthy controls, a model could achieve impressive accuracy by detecting nonspecific slowing or reduced range of motion, that is, by recognizing left hemisphere stroke with a weak arm rather than apraxia. The critical comparison is therefore not apraxia versus healthy controls, but apraxic patients versus neurologically affected patients with comparable weakness, lesion burden, age, and general cognitive impairment but without apraxia. This is precisely the design that allowed earlier kinematic work to show that apraxic deficits were not a mere reflection of left hemisphere damage [2], and any credible validation of the present hypothesis must adopt it. Healthy controls may be included as an additional reference, but they cannot be the primary comparison.

What Distinguishes Apraxic From Elementary Motor Degradation

Group B is defined by the absence of apraxia rather than by the absence of movement abnormality, and its members may show spasticity, intention tremor, or cerebellar ataxia. Each of these produces movement fragmentation, additional velocity peaks, and spatial curvature deviation, which are among the very features proposed here as markers of apraxic execution. The design therefore requires an explicit statement of what is hypothesized to separate the two, and that statement cannot rest on any single kinematic quantity, because at the level of an individual trial the surface signatures overlap substantially.

The discriminating principle proposed here is content dependence. Cerebellar and pyramidal degradation is hypothesized to scale with the mechanical demand of a movement and to be largely indifferent to its symbolic content, so that a spastic or ataxic limb should show a comparable degree of fragmentation, curvature deviation, and velocity irregularity whether the requested movement is a meaningful transitive gesture, a meaningless posture, or the same action performed with the real object in hand. Apraxic degradation is hypothesized to dissociate across exactly those conditions, following the classical pattern in which pantomime is impaired relative to actual tool use and gesture to command is impaired relative to imitation. The primary discriminating quantity is therefore not a kinematic variable measured on a single trial but a within-participant contrast computed across elicitation conditions, for example the difference in trajectory deviation between pantomimed and object-mediated performance of the same action, or between command and imitation of the same gesture. This makes the structure of the task battery part of the measurement itself and requires that every participant in Groups A and B perform matched actions under each elicitation condition.

**Figure 3:**
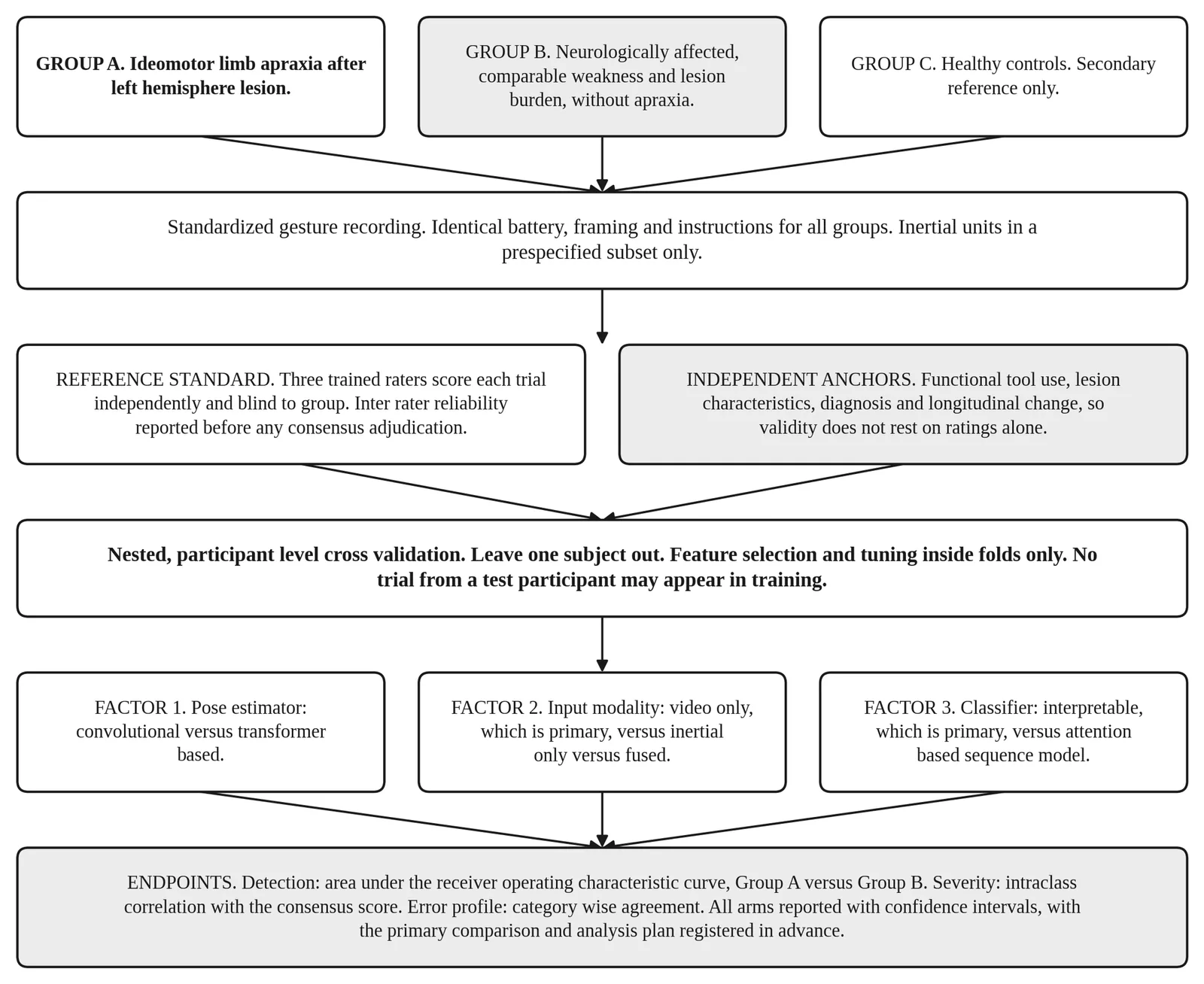
Proposed validation design and prespecified comparison arms Group A versus Group B is the critical comparison. Comparison against healthy controls alone would permit a model to achieve high accuracy by detecting weakness or nonspecific slowing rather than apraxia. Three factors are varied within a single nested, participant level cross validation: pose estimator family, input modality, and classifier family. The video only, interpretable configuration is the primary analysis and the remaining arms are prespecified secondary comparisons.

Three secondary features are proposed to assist the separation and should be tested as covariates rather than assumed. First, intention tremor occupies a comparatively narrow frequency band in the region of three to five hertz and should be identifiable, and where necessary removable, by spectral decomposition of the joint angle time series before submovement counting, whereas apraxic fragmentation is not expected to produce a comparable narrow band peak. Second, spastic movement is characterized by velocity-dependent reduction in peak velocity with relative preservation of the overall shape of the trajectory, so that peak velocity and normalized path curvature should dissociate, whereas apraxic spatial error is expressed in the shape of the trajectory and in limb and hand orientation relative to an imagined target, with peak velocity comparatively less affected. Third, cerebellar dysmetria is expressed predominantly as endpoint overshoot or undershoot followed by terminal corrective submovements, whereas apraxic spatial error typically appears earlier in the movement as an incorrect plane of action or an incorrect hand orientation, so that the position of the deviation within the normalized movement trajectory carries discriminating information. These contrasts are stated as hypotheses. If none of them separates Group A from Group B once weakness and lesion burden are accounted for, the hypothesis is disconfirmed in the stronger form. Figure 3 shows the overall design, and Table 3 summarizes its elements.

**Table 3:** Summary of the proposed validation design The design is offered as a feasible test of the hypothesis. No empirical results are reported and no data have been collected.

Design element	Specification
Groups	Group A: patients with ideomotor limb apraxia after left hemisphere lesion. Group B: neurologically affected patients with comparable weakness, lesion burden, age, and general cognitive impairment but without apraxia. Group C: healthy controls, as a secondary reference only
Critical comparison	Group A versus Group B. Comparison against healthy controls alone would allow a model to succeed by detecting weakness or nonspecific slowing rather than apraxia
Task battery	Standardized gesture production under command and imitation, aligned with a validated instrument, with identical framing and instructions across all groups
Recording	Commodity RGB camera with standardized framing at a minimum of sixty frames per second. Two synchronized views, one frontal and one oblique at approximately forty five degrees, are the recommended default for the validation study; a single frontal view is retained as the minimum deployable configuration and reported as a prespecified secondary comparison. Synchronized inertial units in a prespecified subset only. Acquisition conditions standardized as set out in the acquisition checklist.
Reference standard	Three trained raters scoring each trial independently and blind to group, using a prespecified scoring manual. Inter rater reliability reported before any consensus adjudication, with disagreements retained rather than erased
Additional validity anchors	Functional tool use, lesion characteristics, diagnosis, and longitudinal change, so that validity does not rest on expert ratings alone
Evaluation	Nested, leave one subject out cross validation. All splitting at the participant level. Feature selection and tuning inside folds only. Performance reported with confidence intervals for every arm
Primary endpoints	Area under the receiver operating characteristic curve for detection of apraxia, Group A versus Group B; intraclass correlation between predicted severity and consensus score
Secondary endpoints	Category wise agreement for the error profile; keypoint stability against manual annotation or motion capture in a subset; comparative performance across the factors in Table 4
Ceiling	The model should not be expected to exceed the reliability of the raters who generated its labels. Matching that reliability would already be a meaningful result

Ground Truth and Its Limits

Ground truth would be established by independent scoring of each performance by at least three trained raters using a prespecified scoring manual and a validated instrument, with interrater reliability reported before any consensus adjudication, and with disagreements retained as informative rather than erased. The reference standard is itself imperfect, since it rests on expert judgment that this report elsewhere describes as variable; the appropriate response is not to deny this but to report rater agreement transparently, to treat that agreement as the realistic ceiling for the automated system, and to anchor validity additionally to independent outcomes such as functional tool use, lesion characteristics, diagnosis, and longitudinal change. The model should not be expected to exceed the reliability of the raters who generated its labels; matching that reliability would already be a meaningful result. The treatment of rater disagreement in model training follows from this position and should be specified rather than left to convention. Forcing each trial to a single majority label discards the information carried by disagreement and presents the model with an artificially confident target on precisely those trials that are clinically ambiguous. The approach adopted here is soft label training. The target for each trial is the distribution of ratings assigned by the panel, so that a trial judged impaired by two of three raters is presented as a target of approximately two-thirds rather than as a categorical impairment, and the objective is a cross-entropy computed against that distribution rather than against a hard consensus label. Ordinal severity targets are handled equivalently, either by label distribution learning across the ordinal categories or by regression against the mean rating with the interrater standard deviation carried as a per-trial weight, so that trials on which the panel agreed exert greater influence than trials on which it did not. Trials showing disagreement are retained in both training and evaluation rather than excluded, since excluding them would inflate apparent performance by removing the hardest cases. Model performance is then interpreted against the observed interrater agreement rather than against perfect agreement. Panel reliability, expressed as an intraclass correlation coefficient for severity and as a chance-corrected agreement statistic for the error categories, is reported before any model result and is treated as the realistic ceiling on achievable performance.

Evaluation and Generalization

Given the small samples typical of apraxia research, likely fewer than 100 patients, the modeling approach should favor interpretable classifiers such as random forests or support vector machines over deep networks trained from scratch. Evaluation should use leave one subject out cross-validation, so that every held-out test patient is unseen during training and no frames from a test patient can leak into the training set. All data splitting must occur at the participant level, never at the trial level; feature selection and model tuning must occur within cross-validation folds, not before them; and performance must be reported with confidence intervals. Suitable primary metrics are the intraclass correlation coefficient between the model's predicted severity and the expert consensus score, and, for binary detection, the area under the receiver operating characteristic curve. Several distinct generalization claims should also be separated and tested in order of increasing difficulty: performance on new trials of trained gestures, performance on new patients, performance on gestures not seen in training, performance under different elicitation conditions, and performance with a different camera or at a different site. Generalization to unseen gestures and unseen sites is a substantially stronger claim than generalization to new patients performing the same standardized gestures, and multisite, held out site evaluation would eventually be necessary before any clinical claim. Pre-registration of the analysis plan and transparent reporting of negative results would strengthen the credibility of any positive finding.

Three factors should be varied within this same evaluation framework rather than fixed in advance: the pose estimator family, comparing at least one lightweight convolutional model with at least one transformer- based model; the input modality, comparing video-only, inertial-only, and fused pipelines; and the classifier family, comparing an interpretable model with a prespecified attention-based alternative. The video-only, interpretable configuration is the primary analysis, because it is the configuration that carries the clinical claim, and the others are prespecified secondary comparisons. Because a design of this shape invites multiplicity, the primary comparison and the full analysis plan should be registered in advance, secondary comparisons should be labeled as such in reporting, and effect sizes with confidence intervals should be given for every arm rather than only for the arm that happened to perform best. Table 4 lists these factors, their levels, and the role each plays in the analysis.

**Table 4:** Prespecified experimental factors, levels, and their role in the analysis All factors are varied within the same nested, participant level cross validation. Because this design invites multiplicity, the primary comparison and full analysis plan should be registered in advance and secondary comparisons labeled as such in reporting.

Factor	Levels	Role	Rationale
Pose estimator family	At least one lightweight convolutional model and at least one transformer based model, applied to identical recordings	Secondary	Determines whether estimator choice changes the clinically relevant features. Interchangeability would itself be a useful and reassuring result
Input modality	Video only; inertial only; filter fused video and inertial	Video only is primary; the others secondary	The video only arm carries the clinical claim. The fused arm establishes an upper bound, so that any shortfall of the video only system can be attributed either to measurement quality or to the hypothesis itself
Classifier family	Interpretable model over engineered features; attention based sequence model over the same feature sequences	Interpretable model is primary	Apraxia cohorts are small and attention based models overfit at that scale. The sequence model is admissible only with pretraining or transfer, strong regularization, and participant level evaluation
Generalization tier	New trials of trained gestures; new participants; unseen gestures; different elicitation conditions; different camera or site	Reported separately in order of increasing difficulty	These are distinct claims of increasing strength. Held out site evaluation would be required before any clinical claim

## Discussion

Predicted outcomes

If the hypothesis holds, continuous spatial and temporal features recovered from video would differ significantly between apraxic patients and appropriate comparison groups, reproducing at the level of individual patients the group differences that laboratory kinematic studies have reported. Severity estimates from the model would correlate strongly with expert consensus scores on the reference battery. The error dimension profile the system produces would align with the error types independently assigned by clinicians for the dimensions accessible to level one analysis, with weaker performance expected on the content and conceptual errors that belong to level two.

Refutation is equally specified. If computed kinematic features failed to separate apraxic from control performances once weakness and general brain damage were controlled, or if a model trained on expert ratings failed to generalize to new patients, new raters, or new recording conditions, the hypothesis would be disconfirmed in its stronger form. A particularly informative partial result would be a system that captures spatial and temporal errors but fails on content errors, which would confirm rather than surprise the framework, since content errors are expected to lie largely beyond single gesture kinematics. Anticipating that boundary is part of taking the hypothesis seriously rather than merely advocating for it.

Three technical comparisons carry predictions of their own. Transformer-based pose estimation is predicted to yield more stable distal hand landmarks than lighter convolutional models under occlusion and atypical posture, with the caveat that any such advantage may not propagate to classification once features are aggregated across a trial. Video inertial fusion is predicted to improve the stability of the kinematic features under variable lighting and partial occlusion relative to video alone, and the size of that gain would bound what the video-only configuration gives up. A transformer classifier is predicted to capture sequential and ordering errors better than a model that averages features over a trial, but only if pretraining or pooled data relieve the sample size constraint. At realistic apraxia cohort sizes the simpler interpretable model may well win, and a result in that direction should be reported as such rather than treated as a failure of the framework. Table 5 states each prediction alongside the observation that would refute or bound it.

**Table 5:** Predictions and the observations that would refute or bound them Refutation criteria are stated in advance so that the hypothesis is falsifiable rather than merely advocated.

Prediction if the hypothesis holds	Observation that would refute or bound it
Continuous spatial and temporal features recovered from ordinary video separate apraxic from non apraxic gesture production at the level of individual patients	Features fail to separate Group A from Group B once weakness, lesion burden, and general cognitive impairment are accounted for
Model derived severity correlates strongly with blinded expert consensus on the reference battery	Correlation falls well below the observed inter rater reliability, or the model fails to generalize to new patients, new raters, or new recording conditions
The error profile aligns with clinician assigned error types for the dimensions accessible to level one analysis	Category wise agreement is no better than chance for spatial, trajectory, or temporal dimensions
Content and conceptual errors are captured less well than spatial and temporal errors	This is an expected boundary rather than a refutation. A system that succeeds on level one and fails on level two confirms the framework as stated
Transformer based pose estimation yields more stable distal hand landmarks under occlusion and atypical posture	No difference in keypoint stability, or a difference that does not propagate to classification once features are aggregated across a trial
Video inertial fusion improves feature stability under variable lighting and partial occlusion relative to video alone	No measurable gain, which would strengthen rather than weaken the video only claim
An attention based sequence model captures ordering and sequencing errors better than a model that averages features across a trial	The interpretable model performs as well or better at realistic cohort sizes, which should be reported as such rather than treated as a failure of the framework

Implications

Should the hypothesis be supported even in its bounded form, the consequences would be meaningful. An objective, continuous measure would make apraxia severity comparable across examiners, institutions, and time, which matters both for individual monitoring and for clinical trials, where a reliable quantitative endpoint is often the limiting factor. Because the approach relies on ordinary video, it could extend structured apraxia assessment to settings without a behavioral neurologist, including remote and telemedicine contexts, and could support rehabilitation by giving patients and therapists measurable feedback on gesture production. The continuous features could also serve mechanistic research by offering a finer-grained dependent variable than ordinal scores allow. None of these benefits would require removing the clinician from the assessment; the framing throughout is augmentation, with diagnostic responsibility remaining with the clinician and with the automated output open to override.

Limitations and ethical considerations

The limitations are substantial and define the work required before any clinical claim. First, the approach is proposed here and has not been built or validated; this report offers a hypothesis and a design, not results. Second, the reference standard is imperfect, because it rests on expert ratings whose reliability, though good, is not perfect, and a model can be no more reliable than its labels. Third, markerless pose estimation has known error, particularly for fine finger configuration and hand orientation in depth, under occlusion, motion blur, poor lighting, and low resolution, and abnormal postures in a stroke population may degrade it further; since some apraxic errors are expressed precisely in hand configuration, pose estimation accuracy is a genuine constraint that must itself be validated rather than assumed. Fourth, kinematics describe how a movement occurred but not whether the intended action was correct, so content and conceptual errors lie largely beyond a single gesture kinematic analysis, bounding the stronger form of the hypothesis. Fifth, apraxic performance can be confounded by coexisting hemiparesis, spasticity, proprioceptive loss, ataxia, neglect, tremor, and executive dysfunction, which is why the without apraxia control group is essential and why claims must be guarded against a model that merely detects arm dysfunction. Sixth, apraxia datasets are small while each video yields high-dimensional data, creating substantial overfitting risk that participant-level splitting and honest reporting can mitigate but not remove. Seventh, ideomotor apraxia is heterogeneous across lesion site, etiology, gesture type, and elicitation condition, so a single binary label may obscure real structure. Eighth, good performance on pantomime or imitation does not by itself establish prediction of real-world tool use or daily function, and functional validity should not be claimed unless an independent functional measure is included.

Several further limitations attach specifically to the technical extensions described above. Attention-based models, whether used for pose estimation or for classification, increase computational cost and data requirements relative to lighter alternatives, and the larger estimator variants may not run acceptably on the commodity hardware that the bedside claim assumes. Inertial augmentation introduces sensor placement compliance, per-session calibration, and synchronization between the video and inertial streams, each of which is a practical failure point in a clinical setting; filter-based fusion adds tuning choices that can themselves be overfitted to a small sample. Comparing several estimators, modalities, and classifiers multiplies the analyses and, without prespecification, multiplies the opportunities for a favorable result to arise by chance. Finally, the operationalization of body part as object errors offered above is almost certainly too coarse to stand on its own, and a more adequate treatment may require hand mesh reconstruction or explicit comparison against reference gestures, which remains unresolved.

Ethical development would require attention to consent for video capture and storage, protection of identifiable recordings, the risk that an automated score is granted more authority than its validation supports, and equitable performance across patient groups. A system of this kind should be introduced, if at all, as a measured adjunct to expert judgment, with its limitations disclosed and its outputs open to clinical override, and never as an autonomous diagnosis. Three specific ethical questions arise from this technology in particular and are worth stating rather than leaving to the general framing above. The first concerns disagreement between the clinician and the automated output. The design position adopted here is that the clinical judgment prevails without justification being required, that the automated score is recorded alongside it rather than replaced by it, and that systematic patterns of disagreement are audited rather than discarded, since a recurring divergence in a particular patient subgroup is evidence about the system rather than about the clinician. The second concerns responsibility when an automated score is wrong and contributes to a mistaken assessment. Because the output is a measurement adjunct and not a diagnostic determination, clinical responsibility remains with the assessing clinician, and this should be stated explicitly in any deployment rather than left implicit; the corresponding obligation on the developer is that the validation limits, the populations in which performance was established, and the conditions under which performance degrades are disclosed alongside every output, so that the clinician can weigh the score appropriately rather than inherit an unqualified number. The third concerns consent in a population in which aphasia and cognitive impairment are common, and in which the very deficits under study may compromise the capacity to consent to being recorded. Consent procedures should therefore not rely on written or verbal comprehension alone, should use simplified visual and demonstrative formats appropriate to receptive aphasia, should involve a legally authorized representative where capacity is absent, should treat consent as revocable at any point including during a recording, and should be re-sought rather than assumed if capacity changes over a longitudinal protocol. Video is identifiable in a way that ordinal scores are not, which raises the threshold for all three of these safeguards rather than lowering it.

## Conclusions

Apraxic movement has been measured quantitatively for more than three decades, yet those measurements have stayed in the laboratory, while routine assessment has remained a matter of observation and ordinal scoring. The tools now exist, in markerless computer vision and machine learning, to approximate the informative measurements from ordinary video and to organize them around the error dimensions clinicians already recognize. The hypothesis advanced here is bounded and defensible: that such a system can serve as an objective measurement adjunct for ideomotor limb apraxia, quantifying selected spatial and temporal features and profiling error dimensions, validated against blinded expert consensus and, critically, against a control group of neurologically affected patients without apraxia. It is offered as a testable hypothesis with a concrete validation path and an explicit account of what would refute it. It does not propose to replace the neurologist at the bedside. It proposes to give that examination something the laboratory has long had but the clinic has not: a measure.
